# Analysis of the correlation between biomechanical properties and corneal densitometry in myopic eyes

**DOI:** 10.3389/fbioe.2023.1182372

**Published:** 2023-04-26

**Authors:** Yuwei Zheng, Chao Xue, Jing Wang, Xuan Chen, Xiaohui Wang, Yan Wang

**Affiliations:** ^1^ Clinical College of Ophthalmology, Tianjin Medical University, Tianjin, China; ^2^ Tianjin Key Lab of Ophthalmology and Visual Science, Tianjin Eye Hospital, Tianjin Eye Institute, Nankai University Affiliated Eye Hospital, Tianjin, China; ^3^ The Second Affiliated Hospital of Anhui Medical University, Hefei, China

**Keywords:** corneal densitometry, Corvis ST, small incision lenticule extraction (SMILE), uniaxial tensile test, biomechanic

## Abstract

**Background:** To investigate the correlation between corneal biomechanical characteristics (*in vitro* and *in vivo*) and corneal densitometry in myopia.

**Methods:** The Pentacam (Oculus, Wetzlar, Germany) corneal densitometry (CD) and Corvis ST (Oculus, Wetzlar, Germany) exams were conducted prior to surgery for myopic patients who were intended to undergo small-incision lenticule extraction (SMILE). CD values (grayscale units, GSUs), and *in vivo* biomechanical parameters were obtained. The stromal lenticule was subjected to a uniaxial tensile test to obtain the elastic modulus E *in vitro*. We exam the correlations among *in vivo*, *in vitro* biomechanical characteristics and CD values.

**Results:** In this study, 37 myopic patients (63 eyes) were included. The mean age of participants was 25.14 ± 6.74 years (range:16–39 years). The mean CD values of the total cornea, anterior layer, intermediate layer, posterior layer, 0–2 mm region and 2–6 mm region were 15.03 ± 1.23 GSU, 20.35 ± 1.98 GSU, 11.76 ± 1.01 GSU, 10.95 ± 0.83 GSU, 15.57 ± 1.12 GSU and 11.94 ± 1.77 GSU, respectively. Elastic modulus E (*in vitro* biomechanical indicator) was negatively correlated with intermediate layer CD (r = −0.35, *p* = 0.01) and 2–6 mm region CD (r = −0.39, *p* = 0.00). A negative correlation was also found between 0-2 mm central region CD and *in vivo* biomechanical indicator SP-HC (r = −0.29, *p* = 0.02).

**Conclusion:** In myopic patients, densitometry is negatively correlated with biomechanical properties both *in vivo* and *in vitro*. With an increase in CD, the cornea deformed more easily.

## Introduction

With the expansion of the myopia population in China, the number of refractive surgeries is also increasing year by year (Wang et al., 2020). Corneal biomechanical properties are closely related to multiple aspects of refractive surgery (Ma et al., 2018). Biomechanical inspection can help clinicians exclude patients with corneal ectasia tendencies before surgery and choose personalized surgical methods. For patients who are prone to undergo refractive regression, postoperative medications should be adjusted in a timely fashion (Qi et al., 2017). This means that a deep understanding of corneal biomechanical properties will be very helpful in clinical diagnosis and treatment (Raevdal et al., 2019). At present, corneal biomechanical characteristics can be obtained *in vitro* and *in vivo*, while *in vivo* measurements are more common. Corvis ST (Oculus, Wetzlar, Germany), a biomechanical instrument that has good accuracy and repeatability, is increasingly being used.

As an index of corneal morphology, corneal densitometry (CD, also known as optical density) (Charpentier et al., 2021) was obtained by a high-speed Scheimpflug Pentacam camera (Oculus, Wetzlar, Germany). By quantifying the backscatter light from the cornea, the transparency of each layer, region, and total cornea was measured. The stroma accounts for approximately 90% of the human cornea. The diameter and arrangement of the stromal collagen fibers directly affects the corneal transparency (Espana and Birk, 2020), which can be discribed as CD value (grayscale units, GSUs). The spacing, distribution and direction of the collagen fibers also impacts the biomechanical indicators. At present, knowledge of the relationship between corneal optical indicators and biomechanics is deficient. SMILE surgery provides stromal tissue that can be used for mechanical tensile testing. Therefore, we studied the correlation between biomechanical properties and CD.

Limited research has been performed on the correlation between corneal biomechanics and optical densitometry. The results of Li et al. (2020) showed that the central region CD had a significant correlation with Corvis ST SP-A1. To the best of our knowledge, there have been no studies on the correlation between the *in vitro* elastic modulus and optical densitometry of human corneas. We hope that our study will provide some references for clinical work through analyzing the correlation between biomechanical indicators (both *in vivo* and *in vitro*) and corneal densitometry.

## Materials and methods

### Participants

A retrospective study was conducted of 37 patients (63 eyes) with myopia treated in the refractive center of Tianjin Eye Hospital, Tianjin Medical University from 1 March 2017 to 1 September 2018, including 29 eyes of 16 men and 34 eyes of 21 women. All participants were between 16 and 39 years old, with an average age of 25.14 ± 6.74 years, and the equivalent sphericity (SE) was between −1.5D and −10.0D. All participants underwent routine preoperative examinations, including vision, optometry, slit-lamp microscopy, fundus examination and intraocular pressure (IOP, by Topcon). After SMILE surgery, the corneal lenticule obtained during the operation was subjected to a uniaxial tensile test to measure its biomechanical properties *in vitro*. The exclusion criteria were as follows: a previous history of eye surgery, previous wearing of contact lenses, a history of eye trauma, glaucoma, pterygium and other eye diseases, pregnancy or lactation, rheumatic immune system diseases and abnormal thyroid function. This study followed the principles of the Declaration of Helsinki and was approved by the Ethics Committee of the Tianjin Eye Hospital. All enrolled participants signed an informed consent form.

### Methods

#### Corneal densitometry

The examination was performed by the same skilled technician in one dark room. Participants were told not to move or blink their eyes during the inspection. Twenty-five images for each patient were taken with a Scheimpflug camera, and the machine automatically analyzed the CD values of four circular or concentric ring areas (0–2 mm, 2–6 mm, 6–10 mm, and 10–12 mm diameter). Meanwhile, the machine will layer the cornea according to the depth and measure the CD values of each layer. The anterior layer is 120 μm thick. The posterior layer is 60 μm near the atria, while the intermediate layer is the section between the anterior and posterior layers. The instrument provided the central corneal thickness (CCT) of the participants at the same time.

#### Corvis ST biomechanical analyzer

The examination was performed by the same skilled technician. Participants were told to keep their eyes on the indicator light and not move or blink during the examination. The air pulse will start automatically, and the cornea will go through the process from stillness to depression and then rebound to the physiological state. The Scheimpflug camera automatically took 140 images to record the deformation process. The results of the quality index “OK” were included in the analysis, and the average value was taken from three consecutive measurements. After the test, the instrument automatically analyzed the data and calculated the corneal deformation indicators. Eight commonly used mechanical indices of Corvis ST were selected for research. The indicators are shown as follows.

Eight commonly used Corvis ST indicators.

**Table:** 

Indicators	Description
A1 T	First applanation time (ms)
A1 V	Velocity of the first applanation (m/s)
PD	Peak distance at the largest applanation (mm)
Integrated radius	The integrated sum of the inverse concave radii between the first and second applanation events (/mm)
SP-A1	The stiffness parameter at the first applanation (mmHg/mm)
SP-HC	The stiffness parameter at the largest applanation (mmHg/mm)
DA ratio 2 mm	Deformation amplitude 2 mm away from the apex and the apical deformation

#### Small incision femtosecond laser lenticule extraction (SMILE) surgery

All surgeries were successfully completed by the same experienced chief physician. Levofloxacin 0.5% (Santen, Japan) eye drops were routinely used 4 times a day for 3 days before the operation. Eye drops of obukaine hydrochloride were used for surface anesthesia prior to the operation. A VisuMax femtosecond laser (Zesis Company, Germany) was used for the surgery at frequency 500 kHz, energy 115–130 NJ. The thickness of the corneal cap was 110 μm. The diameter of the stromal lenticule was 6–6.5 mm, and the corneal cap diameter was the lenticule’s diameter plus 1 mm. Laser scanning was first performed under the lenticule, then lateral cutting, and finally scanning was performed above the lenticule. A 3 mm micro incision was made at 12 o’clock, and the base thickness was 10–15 μm. After scanning, the matrix tissue was separated and removed from the cornea and then carefully checked to determine whether the lenticule was integrated.

#### Uniaxial tensile test

After removing the stromal lenticule during the surgery, the 12 o’clock position was marked with gentian violet, and the tissue was immediately placed in preservation solution (Eusol-C, Alchima, Padova). The lenticule was stored in a refrigerator at 4°C for less than 24 h. The stromal tissue was placed on a rubber pad and smoothed out, then we cut the tissue from the top (12 o’clock position) to the bottom into a long strip with a 1 mm width, 6–6.5 mm length (equals to the diameter of stromal lenticule). The test piece was held on the fixture and kept wet with a normal saline water bath. Both ends were fixed with screws. First, the lenticule was preadjusted, and then the tensile force was applied using a mechanical test system (ITBC-50, Kyle Measurement and Control Test System Co., Ltd.) with a speed of 0.01 mm/s uniaxial tension. The stress corresponding to less than 5% of the strain produced was analyzed, and Young’s modulus E was obtained by linear regression analysis. This study used σ = A (e^Be^-1) to describe the relationship between stress and strain and E = B (σ+A) to deduce the linear relationship of the elastic modulus. The results of the elastic modulus corresponded to a stress of 0.02 MPa which resembles the intraocular pressure.

#### Statistical methods

This study used SPSS 26.0 statistical software for data analysis. Because some densitometry and biomechanical values were not normally distrubuted, Pearson and Spearman correlation analysis were used to exam the correlations between variables. Multiple liner regression analysis was used to analysis the relationship between the Young’s modulus E and Corvis ST parameters. *p* < 0.05 was considered statistically significant.

## Results

A total of 37 participants (63 eyes) were included in this study, including 29 eyes of 16 men and 34 eyes of 21 women. The descriptive characteristics are shown in Table 1.

**TABLE 1:** Descriptive characteristics of 37 patients (63 eyes) with myopia.

Variables	Indicators	M±SD/n (%)
Age (year)		25.14 ± 6.74
Sex	Males	16 (43.2)
	Females	21 (66.8)
IOP (mmHg)		15.80 ± 2.08
CCT (μm)		548.16 ± 24.14
Equivalent sphericity (D)		−5.32 ± 1.75
Elastic modulus E (MPa)		2.45 ± 1.72
Biomechanical parameters of Corvis ST	A1 T (ms)	7.19 ± 0.21
	A1 V (m/s)	0.16 ± 0.02
	PD (mm)	5.10 ± 0.20
	Integrated radius (/mm)	8.76 ± 0.99
	SP-A1 (mmHg/mm)	102.42 ± 12.85
	SP-HC (mmHg/mm)	11.80 ± 2.66
	DA ratio 2 mm	4.76 ± 0.64

IOP, intraocular pressure; CCT, central corneal thickness; A1 T, first applanation time; A1 V, velocity of the first applanation; PD, peak distance at the largest applanation; SP-A1, the stiffness parameter at the first applanation; SP-HC, the stiffness parameter at the largest applanation; DA, deformation amplitude.

Generally speaking, the corneal densitometry was higher at the anterior layer. The CD values of each layer and region were shown in Table 2.

**TABLE 2:** Corneal densitometry values of each layer and region.

Location	Corneal densitometry Mean ± SD
Anterior layer	20.35 ± 2.00
Intermediate layer	11.76 ± 1.02
Posterior layer	10.95 ± 0.83
0–2 mm region (total)	15.58 ± 1.13
0–2 mm region anterior	21.90 ± 1.98
0–2 mm region intermediate	12.45 ± 0.72
0–2 mm region posterior	12.05 ± 0.83
2–6 mm region (total)	11.94 ± 1.79
2–6 mm region anterior	16.40 ± 2.86
2–6 mm region intermediate	14.64 ± 3.88
2–6 mm region posterior	10.76 ± 1.18
6–10 mm region (total)	14.03 ± 2.92
6–10 mm region anterior	15.06 ± 3.08
6–10 mm region intermediate	21.13 ± 11.35
6–10 mm region posterior	15.07 ± 5.54
10–12 mm region (total)	16.41 ± 5.66
10–12 mm region anterior	26.29 ± 7.05
10–12 mm region intermediate	20.45 ± 2.74
10–12 mm region posterior	13.52 ± 2.01
Total cornea	15.03 ± 1.24

**FIGURE 1:**
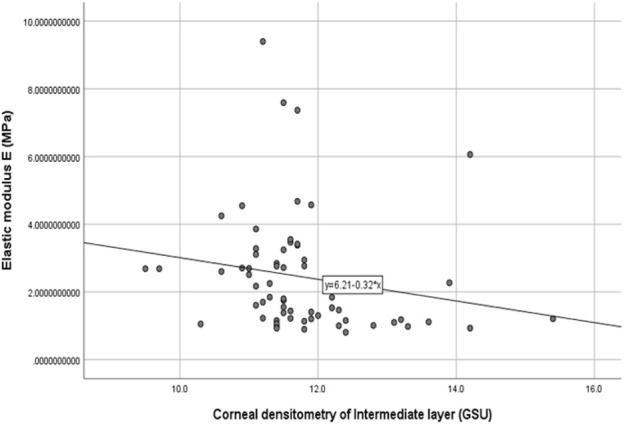
Correlation between the corneal densitometry of intermediate layer and Elastic modulus E (R = −0.35,*p* = 0.01).

**FIGURE 2:**
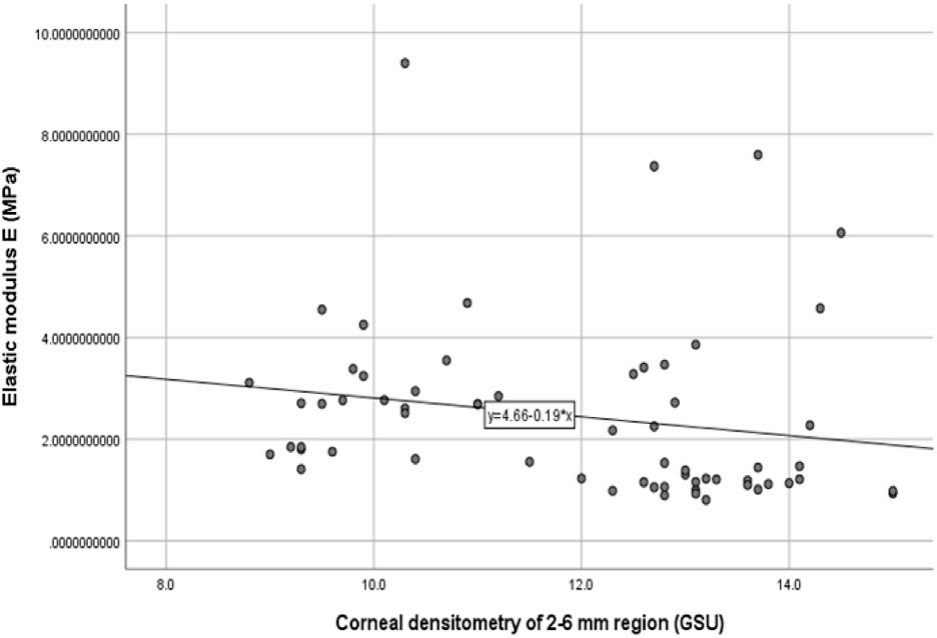
Correlation between the corneal densitometry of 2–6 mm region and Elastic modulus E (R = −0.39,*p* = 0.00).

The stromal lenticule was removed from the central cornea, so we analyzed the *in vitro* elastic modulus E with three layers and the 0–6 mm central region CD values. The correlations between elastic modulus E and CD values are shown in Table 3 and Figure 1 and Figure 2. There was a negative correlation between the elastic modulus E and intermediate layer CD value (r = −0.35, *p* = 0.01). A significant correlation also observed between elastic modulus E and 2–6 mm annulus region CD value (r = −0.39, *p* = 0.00).

**TABLE 3:** Correlation analysis between elastic modulus E and corneal densitometry values.

Corneal densitometry	Elastic modulus E
	(r, P)
Anterior layer	−0.15,0.25
Intermediate layer	−0.35,0.01*
Posterior layer	0.08,0.54
0–2 mm region (total)	0.22,0.08
0–2 mm region anterior	0.09,0.48
0–2 mm region intermediate	0.18,0.15
0–2 mm region posterior	0.26,0.05
2–6 mm region (total)	−0.39,0.00*
2–6 mm region anterior	−0.58,0.00*
2–6 mm region intermediate	0.68,0.00*
2–6 mm region posterior	0.40,0.00*
Total cornea	0.22,0.09

*p < 0.05 is statistically significant.

Correlation existed between multiple biomechanical parameters of Corvis ST and the CD values. A negative correlation was found between 0-2 mm central region CD and *in vivo* biomechanical indicator SP-HC (r = −0.29, *p* = 0.02). The results are shown in Table 4. Most corneal densitometry values which had a correlation were distributed at the 0–6 mm central region of cornea.

**TABLE 4:** Correlation analysis between the biomechanical parameters of Corvis ST and the corneal densitometry values.

Corneal densitometry	A1 T	A1 V	PD	Integrated radius	SP-A1	SP-HC	DA ratio (2 mm)
	(r, P)	(r, P)	(r, P)	(r, P)	(r, P)	(r, P)	(r, P)
0–2 mm region	−0.19,0.14	0.13,0.30	0.32,0.01*	−0.02,0.87	−0.19,0.13	−0.29,0.02*	−0.01,0.93
2–6 mm region	−0.18,0.16	0.19,0.14	0.11,0.39	0.33,0.01*	−0.05,0.69	−0.15,0.24	0.32,0.01*
6–10 mm region	−0.19,0.14	0.30,0.02*	0.17,0.18	0.08,0.56	−0.21,0.10	−0.18,0.15	0.02,0.90
10–12 mm region	−0.06,0.65	0.05,0.69	0.00,0.99	0.18,0.15	0.06,0.66	−0.01,0.92	0.24,0.06
Anterior layer	−0.07,0.61	0.04,0.77	0.13,0.33	−0.04,0.76	−0.07,0.57	−0.17,0.19	0.08,0.56
Intermediate layer	−0.14,0.27	0.10,0.46	0.15,0.23	0.10,0.44	−0.04,0.76	−0.19,0.14	0.17,0.18
Posterior layer	−0.16,0.21	0.22,0.09	0.31,0.01*	−0.07,0.61	−0.14,0.26	−0.24,0.06	0.02,0.88
Total	−0.13,0.31	0.29,0.02	0.18,0.15	0.00,1.00	−0.14,0.28	−0.13,0.30	0.04,0.75

*p < 0.05 is statistically significant.

A1 T, first applanation time; A1 V, velocity of the first applanation; PD, peak distance at the largest applanation; SP-A1, the stiffness parameter at the first applanation; SP-HC, the stiffness parameter at the largest applanation; DA, deformation amplitude.

There was a statistically significant positive correlation between age and *in vitro* elastic modulus E, and A1 V of Corvis ST was also positively correlated with age. CCT had a statistically significant correlation with the many Corvis ST indicators, especially with the corneal hardness parameter SP-HC. Details are shown in Table 5.

**TABLE 5:** Relationship between corneal mechanical indicators and age, CCT, and SE.

	Elastic modulus E	A1 T	A1 V	PD	Integrated radius	SP-A1	SP-HC	DA ratio 2 mm
	(r, P)	(r, P)	(r, P)	(r, P)	(r, P)	(r, P)	(r, P)	(r, P)
**Age**	0.34,0.01*	−0.11,0.38	0.35,0.01*	0.05,0.68	0.22,0.09	−0.24,0.06	−0.13,0.31	0.19,0.14
**CCT**	0.00,0.99	0.18,0.16	−0.32,0.01*	0.11,0.39	−0.36,0.00*	0.64,0.00*	0.31,0.01*	−0.38,0.00*
**SE**	0.25,0.05	−0.03,0.80	0.12,0.37	−0.06,0.65	−0.13,0.31	−0.13,0.31	−0.06,0.66	−0.09,0.47

*p < 0.05 is statistically significant.

CCT, central corneal thickness; SE, equivalent sphericity; A1 T, first applanation time; A1 V, velocity of the first applanation; PD, peak distance at the largest applanation; SP-A1, the stiffness parameter at the first applanation; SP-HC, the stiffness parameter at the largest applanation; DA, deformation amplitude.

Correlation between *in vitro* elastic modulus E and the *in vivo* biomechanical indicators: there was a correlation between the elastic modulus E and the Corvis ST biomechanical indicators. Our team had previously studied and equated the two (Xue et al., 2021). The elastic modulus E of myopia can be calculated from the *in vivo* biomechanical parameters as follows: E = −49.47 + 9.188A1T + 9.946A1 V-0.195DA +0.077SP-HC. Refer to our previous study for the details. Our research had good consistency with the previous results (R^2^ = 0.543). The regression model is statistically significant (F = 2.90, *p* = 0.00).

## Discussion

Previous researcher (Atalay et al., 2021) thought that changes in the corneal mechanical properties preceded any structural lesions. Some corneal diseases, such as keratoconus, have abnormal biomechanical properties prior to morphological corneal dilation. However, with the development of optical technology, the priority of morphological and mechanical changes has become a problem worthy of reconsideration. Recently, using a Pentacam optical densitometry to measure corneal transparency has become increasingly common. Pentacam is an anterior segment analyzer based on a Scheimpflug camera, which has the advantages of fast detection, non-invasiveness and good repeatability. Its CD measurement has been widely used in the early diagnosis of keratitis, corneal dystrophy, keratoconus, Fuchs endothelial dystrophy and other eye diseases in recent years (Guo et al., 2021). The measurement of the CD is of great value for the evaluation of corneal health.

As the main component of the corneal stroma, collagen fiber plays a decisive role in corneal transparency and load-bearing ability (Doughty and Jonuscheit, 2019). Maurice’s lattice theory (Meek and Knupp, 2015) hypothesized that the matrix collagen had the same diameter and that the spacing between fibers was less than one wavelength. This structure cancelled the scattered light, while the transmitted light was not disturbed, making the tissue transparent. The reason for corneal opacity was that the arrangement of the collagen fibers changed (Wilson et al., 2022), and the secondary reflected waves interfered with each other. This hypothesis has some defects, but it can still explain many phenomena. The corneal structure and arrangement of the fibers also determines the mechanical properties of the cornea (Wang et al., 2020). As a heterogeneous and viscoelastic tissue, the cornea has complex mechanical characteristics. The viscosity is closely related to the water content of the stroma (Dackowski et al., 2020), and the elasticity of the cornea is determined by fibers (Goh et al., 2007).

Many diseases, especially in the early stage, have an abnormal CD and biomechanical properties at the same time. Shen et al. (2019) found that the CD value may be related to the severity of keratoconus and negatively related to the SP-A1 of Corvis ST. Patients with Fabry disease have increased CD and abnormal biomechanical properties (Guo et al., 2021), but there has been no report of any correlation analysis between the two. At present, the finite element model that simulates the mechanics and optics of the human cornea needs to be improved. The refractive index of the cornea and various mechanical properties have not been integrated into the model, so the predictability of the results needs to be improved. By discussing the relationship between CD and biomechanical characteristics, we hope that our study will provide references for the optimization of corneal mechanics and optical models.

The development of SMILE surgery not only provides a better refractive correction method for myopia patients but also provides an opportunity for *in vitro* biomechanical research into the cornea. As a viscoelastic material, the Young’s modulus of the cornea is not invariable, and the values reported in previous studies were quite different. This difference may be partly due to the non-linear elasticity of the cornea. The materials used in previous studies were cadaver eyes or diseased corneal tissue (Zeng et al., 2001), which can affect the accuracy of the results.

The materials and experiments on the matrix lens in our study were completed within 24 h after the surgery. The elastic modulus corresponding to 0.02 MPa was selected as the result of the elastic modulus E because the stress was similar to the value of the physiological intraocular pressure. Our results showed that there was a negative correlation between the elastic modulus E and CD of the intermediate layer (r = −0.35, *p* = 0.01). The CD of the anterior layer measured by Pentacam corresponds to the uppermost 120 μm. The posterior layer is the 60 μm cornea next to the anterior chamber, and the intermediate layer is the part between the two layers. This layer was exactly the area corresponding to the matrix tissue removed by surgery. The higher the CD in this layer is, the more irregular the arrangement of the collagen fibers, and a certain level of decline in mechanical properties may also occur. A negative correlation also existed between the 2–6 mm region CD and the elastic modulus E.

An *in vitro* tensile test can obtain the stress–strain curve of the cornea, which is considered to be the gold standard of mechanical measurements (Spiru et al., 2017). However, this test is destructive and cannot be widely applied, so *in vivo* biomechanical measurements are more feasible in the clinic.

At present, the Corvis ST has been widely used in the preoperative examination before refractive surgery. In the correlation analysis between CD and the *in vivo* biomechanical parameters, CD was related to many *in vivo* biomechanical indicators, including A1 V, PD, integrated radius, SP-HC, and DA ratio 2 mm. The related areas were mostly concentrated in the central 0–6 mm region. SP-HC is the index of corneal hardness, which means the pressure at the maximum corneal compress divided by the displacement. PD refers to the distance between the two highest points on corneal rhinometry and the temporal side at the maximum depression. A stiffer cornea has a larger SP-HC and a shorter PD. In this study, SP-HC was negatively correlated with the 0–2 mm central region CD, and PD was positively correlated. This suggested that there is a negative correlation between corneal hardness and CD. This relationship also exists in keratoconus patients (Shen et al., 2019). In participants with myopia, we assumed that the irregular arrangement of collagen fibers may be the reason for the CD increase. Meanwhile, this irregular structure may also reduce the mechanical properties of the cornea.

CCT did not correlate with the elastic modulus E *in vitro* but was correlated with the Corvis ST indicators A1 V, integrated radius, SP-A1, and SP-HC. This is consistent with previous studies (Wang et al., 2017). The elastic modulus E refers to the stress applied on the specimen, so it is not affected by CCT naturally. However, the Corvis ST indicators was greatly affected by CCT. The thicker the cornea is, the higher the corneal stiffness and the smaller the shape variability. Therefore, we can conclude that the elastic modulus E *in vitro* is not affected by corneal thickness. But the influence of CCT should be considered when referring to *in vivo* biomechanical parameters.

The results of our study suggested that there was a statistically significant positive correlation between age and *in vitro* elastic modulus E and a positive correlation between age and the Corvis ST indicator A1 V. With increasing age, the corneal collagen fibers become thicker, the fiber spacing decreases, and the glycosaminoglycan content in the extracellular matrix decreases, resulting in an increase in corneal stiffness and a decrease in viscosity (Blackburn et al., 2019). The increase in collagen cross-linking caused by aging may be the reason for the improvement in corneal mechanical properties.

A limitation of this study was that the data included were limited; only 63 eyes were enrolled from young people aged 16-39. Because it was a retrospective study, comprehensive corneal biomechanical (CBI) data were lacking. The physical examination of this study was carried out between 8 a.m. and 5 p.m., and there was no fixed examination time, so the results may have systematic variations. However, considering that the research target was the correlation analysis, our results still have good reliability.

In conclusion, there is a certain degree of correlation between corneal densitometry and biomechanical parameters. The higher CD of myopic participants indicates a more irregular arrangement of collagen fibers, which means that their corneal mechanical properties may decline. Hospitals lacking biomechanical instruments can pay appropriate attention to the changes in CD. Research on CD has been increasing recently. However, there are still many problems that need to be studied further and explored in more depth, such as whether the content of glycosaminoglycan in the cornea affects CD value, will CD value of myopic patients differ from normal people, and so on.

## Data Availability

The raw data supporting the conclusion of this article will be made available by the authors, without undue reservation.
